# Textile Supplies for Hospitals

**Published:** 1916-04-15

**Authors:** 


### TEXTILE SUPPLIES FOR HOSPITALS.

### IX.—Cotton Uniforms.

The existence of different grades among the staff, and the necessity for distinguishing one grade clearly from another, lead to the employment of more kinds of washing uniforms than would otherwise be required. Variety is cultivated for the sake of appearances more than for other reasons; the intrinsic requirements being similar in the uniforms of all ranks. What is expected from any of them is that they shall provide a little warmth, wear for as long as possible, and look as neat as may be at all stages of their useful career. While the uniforms of nurses are ordinarily expected to look superior to those of servants, and the uniforms of sisters superior again to those of nurses, it is not generally convenient to allow for any very marked disparity in price. Distinction is sought, therefore, in other ways, and there are abundant means of providing it without seriously swelling the bill.

Natural reasons ordain that uniforms intended for a weekly visit to the wash-tub should be either cotton or linen. Wool is definitely less suitable, and silk is debarred by its cost. Linen, although costing more than cotton, is worth more; its surface soils less readily from contact with dust, and its appearance is in its favour for wear by the higher rank. There are, however, disadvantages attaching to dyed linen, for its comparatively woody fibres do not take up dye so readily or retain it so well as does cotton. For this reason, when it is desired to weave in a name in colour in an undyed linen towel, for example, it is the almost invariable practice to weave the name not in coloured linen but in dyed cotton yarn.

### Cloths, Thick and Thin.

In any case coloured linens are not procurable in the wide range of patterns and designs in which cottons are made, and perforce cotton is the staple wear. The cottons in regular use for hospital uniforms are palpably of different thicknesses and weights. Although warmth is only an incidental consideration it may be observed that in plain cotton goods "warmth" and "weight" are nearly interchangeable terms. For reasons that have been previously advanced it is possible to bring in comparatively heavy cottons of coarse construction and light fine goods at approximately the same price per yard of cloth. Were they bought by avoirdupois the difference in cost would be remarkable, but in buying by the yard one and the same price commands a choice of heavier goods or of finer and lighter ones. The fine yarns, of which the latter are made, are more expensive than coarse yarn, but less weight of them is required. "Wear" and "weight" are by no means synonymous terms, and it is every-day experience that thin fabrics survive as long, or longer, than thicker ones. No invariable rule can be laid down upon the one hand or the other, but it is important to remember that the advantage in wearing power does not essentially reside in weight or substance.

The weight of washing fabrics, as of other cottons, is often deceptively increased by the use of sizing materials upon the yarns before the cloth is woven, or by treatment in finishing the cloth. Not only the weight, but the apparent fineness and closeness of the goods are enhanced, and they are given a sharp touch which is suggestive of greater durability than is natural to the article. This added weight is lost in washing, and an approximate idea of the extent of the weighting can be formed by weighing a sample before and after boiling. Repeated boilings in plain water will eventually remove the added size, or it may more effectually be extracted by boiling the sample in a weak solution of soda, steeping it in weak acid, rinsing, and drying. A caution against false inferences has to be given. The purest of cotton in a condition of bone-dryness weighs some 8 per cent. less in that state than after it has had an opportunity of regaining its normal degree of moisture. Cotton dried in an oven recovers 7.83 per cent. of water from the atmosphere, and is then in its conventionally correct condition. Further, it has been found that repeated boilings in water cause natural cotton to lose about 2 per cent. of its weight, and these losses have to be allowed for in drawing deductions from a test.

### Questions of Colour.

The durability of cotton uniforms in use is not contingent solely upon the time taken in wearing into holes. The permanence of the colour is a factor no less than the durability of the fabric, and this cannot be judged from superficial evidences in advance. On the whole, bright colours are more prone to fade than dull ones under the influence of sunlight, but in indoor uniforms fading due to light is only one consideration, and not the most important. Fastness to the alkalies present in soap, and to the acids of perspiration and to the mechanical action of the wash-tub, is of equal consequence. The cheapest available dyes do not answer to the several requirements, and the more expensive ones are only of varying suitability, so that careful choice has to be made between them. The dyes and the colours that can be relied on for good service under the exacting conditions of use are few.

The objection to using pure white uniforms is an economical one, based on the frequency with which these must be washed in order to look seemly. The introduction of colour along with white in the fabric breaks the uniformity of the surface and obscures the presence of the slight amount of soiling that occurs in the wear of one week. Plain-dyed uniforms of self colour are open to somewhat

Note.—Previous articles in this series appeared on December 18, 1915, and January 8 and 22, February 5 and 19, March 4 and 18, and April 1, 1916. The next article will treat of "Linen Goods."

the same objection as plain white, albeit in a modified form. A stain makes itself much more evident upon a surface of self colour than upon one broken into lines. A natural compromise in the circumstances is the simple colour-and-white of which, in one pattern or another, most washing uniforms are made; some approximating to pure white, others to a self colour, according to the proportion of colour used in them.

### Dyes and Prints.

The types of fabric in common use are three: (1) yarn-dyed, in which the thread is coloured before being woven; (2) piece-dyed in self colour; (3) printed. In yarn-dyed goods a slight inequality of colouring is less perceptible and important than in self-coloured cloths, and the method of dyeing adopted generally secures a good penetration of the material by the dyestuff. In piece-dyed goods of the heavier varieties it is not unusual to find the core of the yarn less deeply coloured than the exterior, and this is a disadvantage when the interior fibres become exposed by wear. Generally, a greater weight of dyestuff is deposited on the goods in yarn-dyeing than in piece-dyeing, and hence the colour tends to stand better in wear. Printing is a process performed after weaving, enabling plain undyed cloth to be decorated in any of numerous colours and designs at a relatively low expense. Printing is usually done on light calicoes, which are passed over a cylinder and under a roller engraved in low relief with the pattern selected. The grooves of the roller are filled with a thickened paste of dyestuff similar to that used in yarn or piece-dyeing. The paste is transferred to the surface of the calico, which it penetrates to some depth. The colour is fixed upon the fibre by steam-heating, after which the thickening agents used for convenience in printing are washed out of the fabric. Printing imitates more or less deliberately the effects that can be alternatively got by weaving with coloured yarn, and the best printing can be made to last as long as the fabric on which the work is done. A lilac colour is recognised generally as best for hospital use, and a variety of different appearances are obtained by varying the closeness of the printed lines. The advantage of printed over woven patterns is more conspicuous in light goods than in heavier ones, and although good printing is preferable to poor dyeing, the heavier cottons for uniforms are mainly woven from coloured yarn.

At equal or slightly higher prices, Oxford shirtings are preferred by some managers to prints. This cloth is identified with a particular check pattern of red or blue checks, formed by four threads of coloured yarn separated by ten or twelve white threads in the weft and warp. A comparatively soft weft is used to give the Oxford shirting its characteristic softness. In the somewhat similar Harvard shirting, a slightly different structure is adopted in weaving, more of the hard warp threads are brought to the face of the cloth, and softness of touch is less considered.

The Regatta twill is a blue cloth diversified by stripes of blue-grey. The warp is laid out with six or so ends of blue yarn, followed by an equal number of white threads, and in weaving this with a solid blue weft, the white intervals are toned to grey. The Galatea differs from the Regatta slightly in its weave, and in it, as well as in the plain drill, a preponderance of warp is thrown upon the face of the cloth. The articles all possess good reputations, although these have been jeopardised by the production and sale of goods with inferior dyes and excessive sizing. The best varieties are unexcelled for their purpose, but there is no protection in their mere names, which are applied as freely to poor stuff as to good.

Zephyr is a name covering almost any light-coloured cotton woven with a plain ground. Zephyrs are customarily lighter in weight than the fabrics that have been named, finer in construction and more limp in character. They are made in innumerable varieties of self colour, stripes and checks, and sometimes with cords of thicker yarn forming an extra feature. Zephyrs, in short, present one means of clothing the higher grades in palpably finer garments than the remainder without incurring an appreciable excess of cost.

### Superior Cottons.

The mercerised cottons offer some substitute for dyed linen. They have more to recommend them than their mere prettiness, and it is a mistake to dismiss them as poor relations of silk. Normally, a good class of cotton is used for mercerising, because poor qualities do not yield results commensurate with the expense. The strength of the cotton is very much increased by the process of mercerising, and so is the affinity of the fibre for dyestuff. The effect of the process is visible chiefly in the lustre that is lent to the goods, but the changes induced are radical ones. The physical constitution of the ultimate fibres of the cloth is changed, and so is the composition of their native cellulose. In undergoing the process of mercerising, cotton cloth is run in continuous length into a strong caustic lye, maintained at a low temperature. The immediate effect of immersion is a violent contraction of width, and the cloth, which had hitherto been opaque, becomes of a gelatinous translucency. The edges of the cloth are mechanically gripped, and the fabric is pulled out to its original width. The alkali is washed out with water, and after being passed through a neutralising bath of acid, the cloth is dried. The process is worked on a large scale and economically, and there is no difficulty in obtaining mercerised cottons suitable in colouring and design for hospital use. Some of the best examples are sold under fancy names, the proprietary marks of manufacturers who guarantee the permanence of the colouring used. The treatment adds somewhat to the cost, but the addition is accompanied by a gain in suitability, and good-class mercerised cotton is certainly entitled to mention as a practicable substitute for coloured linen.

